# The effect of osteoarthritis on functional outcome following hemiarthroplasty for femoral neck fracture: a prospective observational study

**DOI:** 10.1186/s12891-015-0767-z

**Published:** 2015-10-16

**Authors:** Christoph Kolja Boese, Benjamin Buecking, Christopher Bliemel, Steffen Ruchholtz, Michael Frink, Philipp Lechler

**Affiliations:** Department of Trauma, Hand and Reconstructive Surgery, University of Giessen and Marburg, Baldinger Straße, 35043 Marburg, Germany; Department of Orthopaedic and Trauma Surgery, University Hospital of Cologne, Joseph-Stelzmann-Straße 9, 50931 Cologne, Germany

**Keywords:** Hemiarthroplasty hip, Femoral neck fracture, Osteoarthritis, Functional outcome, Hip arthroplasty

## Abstract

**Background:**

The influence of pre-existing radiographic osteoarthritis on the functional outcome of elderly patientents with displaced intracapsular fractures of the femoral neck treated by hemiarthroplasty is unclear.

**Methods:**

We prospectively examined the impact of pre-existing osteoarthritis on the functional outcome of 126 elderly patients with displaced intracapsular fracture of the femoral neck treated by hemiarthroplasty.

**Results:**

The mean age of the cohort was 82.7 years. At 12 months, we observed no statistically significant differences in the Harris hip score (*p* = 0.545), the timed up and go test (*p* = 0.298), the Tinetti test (*p* = 0.381) or the Barthel Index (*p* = 0.094) between patients with Kellgren and Lawrence grades 3 or 4 osteoarthritis, and patients with grades 0 to 2 changes. Furthermore, there were no differences in complication or revision rates.

**Conclusions:**

Our findings challenge the hypothesis that pre-existing osteoarthritis is a contraindication to hemiarthroplasty in elderly patients with femoral neck fracture.

## Background

The best strategy for the treatment of displaced femoral neck fracture in elderly patients remains unclear. Because of high revision rates, the concept of reduction and internal fixation has been superseded by endoprosthetic replacement of the femoral head and neck [1]. Total hip arthroplasty (THA) and hemiarthroplasty are now established techniques after femoral neck fracture; each has specific advantages and disadvantages. Whereas THA provides optimal biomechanical and tribologic outcomes [2], in frail elderly patients there have been reports of significantly higher blood loss and duration of surgery [3], as well as possibly higher rates of post-operative dislocations [4, 5] and general complications [6, 7]. In contrast, hemiarthroplasy is reportedly associated with less operative trauma [8] and a lower post-operative dislocation rate [4]. Pre-existing radiographic osteoarthritis of the affected hip is considered by some to be a contraindication for hemiarthroplasy, because of the risk of acetabular erosion and the subsequent need for revision [9]. Given the high prevalence of radiographic osteoarthritis of the hip in elderly individuals [10], this substantially limits the clinical applicability of hemiarthroplasy in the treatment of femoral neck fracture.

We examined the influence of pre-existing radiographic osteoarthritis of the hip on the short-term functional outcome after hemiarthroplasy for displaced femoral neck fracture in elderly patients.

## Methods

We prospectively enrolled 126 consecutive elderly patients (age >60 years) with unilateral femoral neck fracture treated by hemiarthroplasty at a single tertiary trauma referral center (university hospital).

The inclusion criteria were:

- Age >60 years
- Unilateral femoral neck fracture
- Surgical treatment by hemiarthroplasty
- Written informed consent by participant or guardian

The exclusion criteria were:

- Incomplete medical or radiological documentation
- High-energy trauma
- Multiple injured patients
- Pathological fracture secondary to malignant disease
- Pre-existing abnormality of the hip anatomy due to trauma or congenital disease
- Undisplaced femoral neck fracture
- Declined participation in the study by participant or guardian

Orthopedic surgeons experienced in the management of trauma performed all procedures. The hip joint was visualized using an anterolateral approach. All patients received the same acetabular component (Bipolar, Zimmer, Inc., Warsaw, IN, USA). The femoral component was inserted with a third generation cementation technique (Palacos®, Heraeus Medical GmbH, Wehrheim, Germany). In 123 cases a standard stem was used (Original M. E. Müller® straight standard stem with a neck-shaft-angle of 135°, Zimmer, Inc.), but in two cases a high offset version was inserted (Original M. E. Müller® straight lateral stem with a neck-shaft-angle of 135°, Zimmer, Inc.). In one patient a nickel-free straight femoral component was used (Smith & Nephew, London, UK). Post-operatively, all patients were treated according to a standardized protocol, including full weight-bearing mobilization on the first post-operative day. The extent of pre-existing osteoarthritis of the affected hip was assessed on pre-operative radiographs independently by two orthopedic surgeons (C.K.B. and P.L.) according to the Kellgren and Lawrence grade (Table 1). Disagreement was resolved through consensus. To identify the influence of pre-existing osteoarthritis on the primary and secondary outcome measures, patients were grouped according to the extent of radiographic osteoarthritis:

**Table 1:** Baseline characteristics of patients treated with hemiarthroplasty for displaced intracapsular fractures of the femoral neck

	All patients (*N* = 113)	Osteoarthritis grade^a^ 3–4 (*N* = 31)	Osteoarthritis grade^a^ 0–2 (*N* = 82)	*p*-value
Age in years (mean ± SD)	82 ± 7	83 ± 6	82 ± 8	0.709
Gender				
female	87 (77 %)	20 (65 %)	67 (82 %)	0.078
male	26 (23 %)	11 (35 %)	15 (18 %)	
ASA Score (mean ± SD)	3.0 ± 0.5	3.0 ± 0.5	2.9 ± 0.4	0.429
II	19 (17 %)	4 (13 %)	15 (18 %)	
III	81 (72 %)	23 (74 %)	58 (71 %)	
IV	13 (12 %)	4 (13 %)	9 (11 %)	
Pre fracture Barthel Index (mean ± SD)	77 ± 28	73 ± 33	79 ± 26	0.312
MMSE on admission	20 ± 9	19 ± 9	21 ± 9	0.286
27–30 (normal)	35 (31 %)	7 (23 %)	27 (33 %)	
20–26 (mild dementia)	39 (35 %)	10 (32 %)	30 (37 %)	
10–19 (moderate dementia)	21 (19 %)	9 (29 %)	12 (15 %)	
< 10 (severe dementia)	18 (16 %)	5 (16 %)	13 (16 %)	

^a^Osteoarthritis was graded according to Kellgren and Lawrence

– absent, mild or moderate radiographic osteoarthritis (Kellgren and Lawrence grades 0 to 2);
– severe radiographic osteoarthritis (Kellgren and Lawrence grades 3 to 4).

**Fig. 1:**
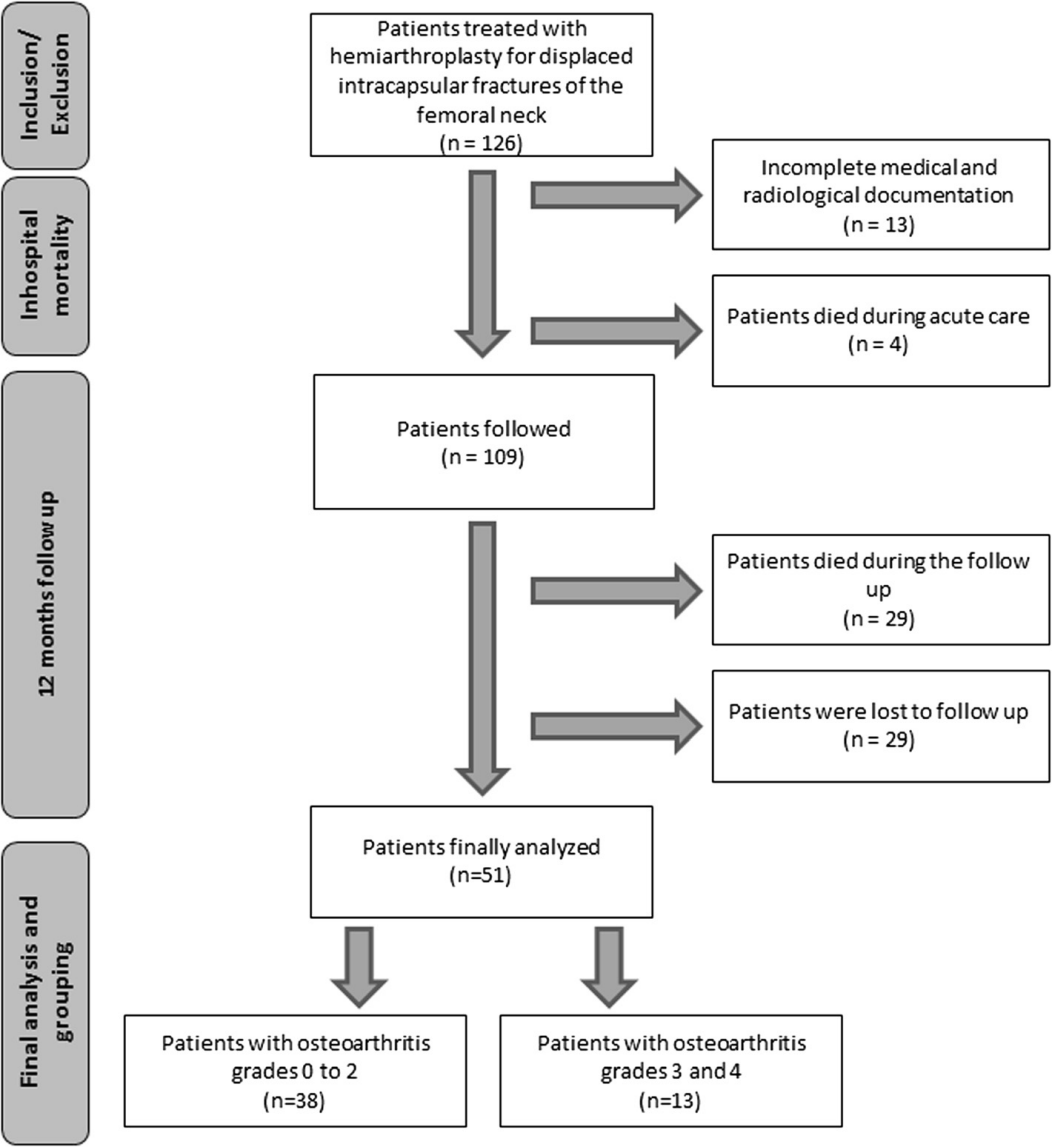
Flow chart of the study

Outcome was assessed 12 months post-operatively in our outpatient clinic or, if patients were unable to attend, in their homes (Fig. 1). The Harris hip score (HSS) was the primary outcome measure, and the timed up and go (TUG) test and Barthel Index were secondary outcome measures. Patient survival, surgical complications and the need for revision surgery were also recorded.

### Statistical analysis

For descriptive analysis, absolute mean values and standard deviations are reported. The distribution of data was assessed for normality using the Kolmogorov-Smirnov test. Comparisons of normally distributed data were undertaken using Student’s *t*-test; the Wilcoxon rank-sum test was used for non-normally distributed data. Adjustment for potentially confounding variables (age, sex, American Society of Anesthesiologists [ASA] physical status score, mini-mental state examination on admission and pre-fracture Barthel Index) was performed by multivariate regression analysis. The limit for statistical significance was set at *p* = 0.05. Data were stored in a database (FileMaker Inc., Santa Clara, CA, USA), and analysis was performed using the SPSS statistics package (Statistical Package for the Social Sciences version 22, IBM Corporation, Armonk, NY, USA) and Excel 2010 for Microsoft Windows (Microsoft Corporation, Redmond, WA, USA).

### Power analysis

Sample size calculation was performed using the nomogram described by Altman. We set the power at 80 % and the significance level at 0.05. The clinically relevant difference in the primary outcome measure (HSS) was set at 16 points. With a standard deviation of 20 points, a standardized difference of 0.8 was calculated, resulting in a minimum sample size of 50 patients.

### Ethics

The study was performed according to the Declaration of Helsinki and its design was approved by the local ethics committee (Ethikkommission des Fachbereichs Medizin der Philipps Universität Marburg, AZ 175/08). Written informed consent for participation in the study was obtained from participants, or guardians. Written informed consent to publish anonymised clinical data was obtained.

## Results

**Fig. 2:**
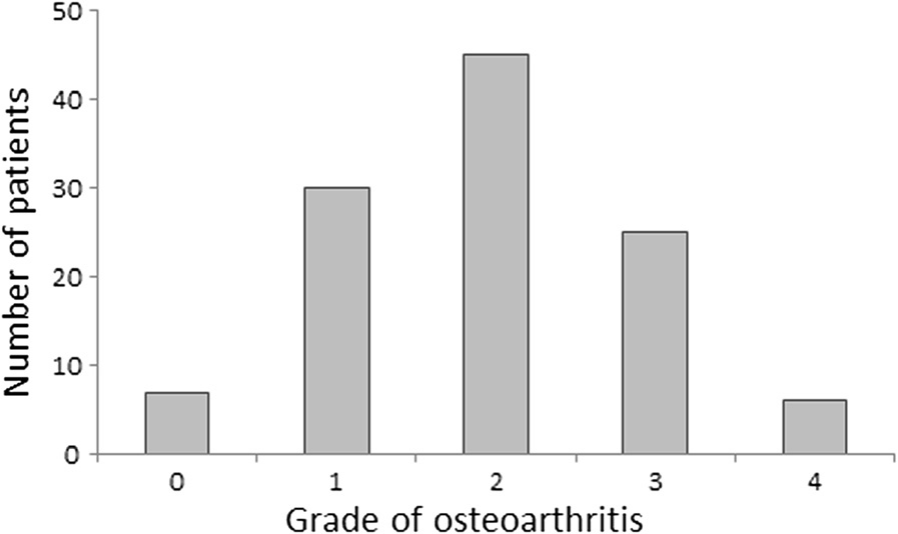
Distribution of the preexisting grades of osteoarthritis. Osteoarthritis was graded according to the Kellgren and Lawrence classification

The baseline demographic and clinical characteristics of the cohort are given in Table 1 and the distribution of the Kellgren and Lawrence grade of osteoarthritis is shown in Fig. 2. We found no significant association between the pre-existing grade of osteoarthritis and the primary and secondary functional outcome measures. Twelve months post-operatively, there were no statistically significant differences between the groups in terms of the HSS as the primary outcome measure (*p* = 0.545), or the TUG (*p* = 0.298) or Tinetti tests (*p* = 0.381) or Barthel Index (*p* = 0.094) as secondary outcome measures (Table 2). These findings were confirmed after adjustment for confounding variables (Table 3). Ten surgical complications were recorded (7.9 %): three cases of post-operative hematoma, two cases of deep infection, two post-operative dislocations, one wound dehiscence and one peri-prosthetic femoral shaft fracture. We found no differences in the frequency of surgical complications or need for revision surgery between the groups. Last, no patient required revision surgery due to the development of acetabular erosion during the follow-up period.

**Table 2:** Functional outcome at 12 month follow up of patients treated with hemiarthroplasty for displaced intracapsular fractures of the femoral neck

	All Patients (*N* = 51)	Osteoarthritis grade^a^ 3–4 (*N* = 13)	Osteoarthritis grade^a^ 0–2 (*N* = 38)	*p*-value
Harris Hip Score	69 ± 21	65 ± 20	70 ± 21	0.545
Timed Up and Go				
Possible (%)	40 (80 %)	9 (75 %)	31 (82 %)	0.686
Time needed (seconds)	28 ± 22	35 ± 27	26 ± 20	0.298
Tinetti Test	17 ± 10	15 ± 9	17 ± 10	0.381
Barthel Index	69 ± 32	61 ± 25	72 ± 34	0.094

^a^Osteoarthritis was graded according to Kellgren and Lawrence

**Table 3:** Influence of osteoarthritis^a^ on functional outcome at 12 month follow up adjusted for gender, age, ASA-Score, MMSE and pre fracture Barthel Index

	Osteoarthritis			
Patients’ functional outcome	B	β	95 % CI of B	*p*-value
Harris Hip Score	2.611	0.054	−12.638; 17.859	0.731
Timed Up and Go	1.196	0.023	−15.708; 18.100	0.886
Tinetti Test	3.029	0.136	−2.442; 8.499	0.270
Barthel Index	3.421	0.045	−13.671; 20.513	0.688

^a^Osteoarthritis was defined as grade 3 or 4 osteoarthritis according to Kellgren and Lawrence

### Case presentation

**Fig. 3:**
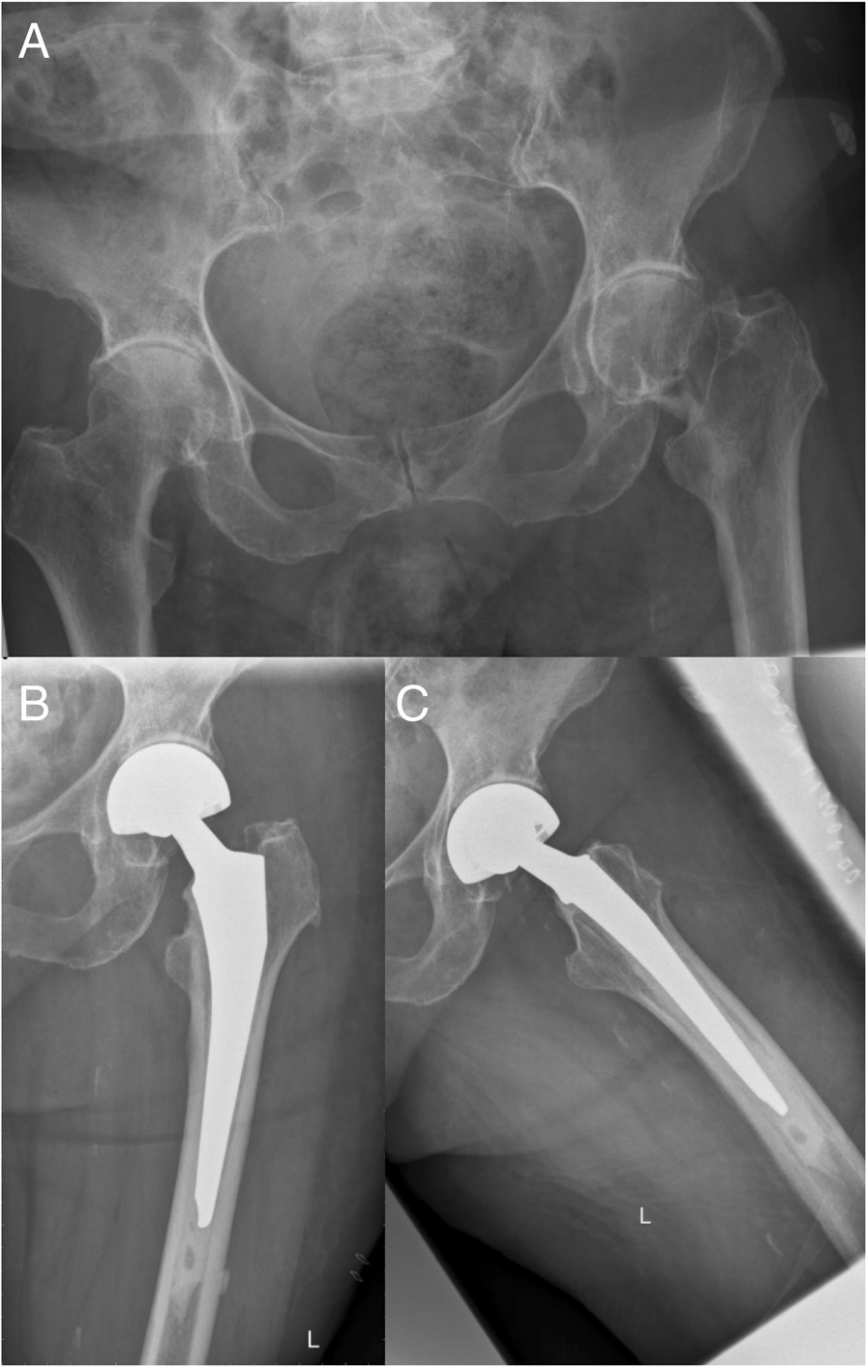
Radiographic documentation of an illustrative case assigned into group 2. **a** Anteropostorior pelvic radiograph depicting a displaced unilateral intracapsular femoral neck fracture (Pauwels 3, Garden IV) of the left hip following a simple low energy fall. The preexisting radiographic osteoarthritis of both hips was graded 3. **b** and **c** Postoperative radiographic documentation of the implanted hemiarthroplasty in two planes

An illustrative case of a patient presenting with a displaced unilateral intracapsular femoral neck fracture (Pauwels 3, Garden IV) of the left hip following a simple fall is given (Fig. 3a). With bilateral radiographic osteoarthritis grade 3, the patient was assigned into group 2. No relevant preexisting pain at the injured or the contralateral hip was reported.

Within 24 h after the trauma, the fractured femur was trated by implantation of a cemented hemiarthroplasty. Postoperatively, the patient was monitored over 24 h at an intensive care unit and mobilized full weight bearing from the first postoperative day. Drainages were removed at the second postoperative day, and radiographs of the injured hip documented the operative result (Fig. 3b and c). The patient was transferred to a specialized geriatric trauma rehabilitation centre 8 days following the operation. 3 ½ weeks after the trauma, the patient was discharged to the care of her family physician in good condition. At the final follow up at 12 months postoperatively, the patient achieved a HSS of 84, a Tinnetti test result of 24, and the time up and go test was performed in 15 s.

## Discussion

Femoral neck fracture accounts for half of proximal femoral fractures and remains a key challenge to the orthopedic surgeon and health care systems [11]. While femoral head-preserving internal fixation is effective in undisplaced fractures and younger patients, the disruption of the blood supply of the femoral head in displaced fracture in the elderly favors the endoprosthetic replacement of the femoral head [12]. Under these circumstances, THA and hemiarthroplasy are competing surgical options for the restoration of hip anatomy and function [4]. Both are highly standardized procedures that allow immediate post-operative mobilization; however, a number of specific advantages and disadvantages of each have been reported [4]. While hemiarthroplasy is reportedly associated with shorter operation time, reduced blood loss, better cost-effectiveness and lower post-operative luxation rates compared with THA, the majority of recent meta-analyses suggest that the latter may provide better functional outcomes and lower reoperation rates following femoral neck fracture [4–6, 9]. Nonetheless, the pre-traumatic patient-specific factors determing mid- and long-term outcomes remain to be clarified [4–6, 9]. The existence of radiologic signs of osteoarthritic degeneration of the affected hip joint has been associated with the risk of the development of acetabular erosion and impaired function following hemiarthroplasty [13], and radiologic evidence of acetabular erosion has been reported to be a major cause for revision surgery [9, 14]. To the best of our knowledge, there has been no systematic study of the implications of the grade of pre-existing radiologic osteoarthritis on the functional outcome or revision rates following hemiarthroplasty for femoral neck fracture in elderly patients.

Just over a quarter of patients in our series (27.4 %) showed showed signs of severe osteoarthritis (Kellgren and Lawrence grade 3 and 4) on pre-operative radiographs, in broad agreement with epidemiological studies on the prevalence of hip osteoarthritis in elderly patients [10] and a recent report on the prevalence of osteoarthritis of the hip in patients with trochanteric fractures [15]. Despite being adequately powered, our study detected no statistical significant differences between elderly patients with absent, mild or moderate radiologic signs of osteoarthritis and those with severe pre-operative osteoarthritis in terms of the functional outcome or revision rates. Our data therefore support the observations by previous authors who reported low overall conversion rates following hemiarthroplasty for femoral neck fracture due to acetabular erosion or other reasons [16–18]. This could be explained by the overall poor agreement between radiological signs of hip osteoarthritis and symptoms [19], as well as the relatively low biomechanical stress and the limited functional demands made of the prosthesis in the majority of elderly patients who sustain these injuries. Interestingly, van den Bekerom et al. defined osteoarthritis of the hip among the exclusion criteria for their recent analysis of the natural history of a large series of patients receiving hemiarthroplasty for displaced intracapsular femoral neck fracture [20]; however, the authors did not provide detailed information regarding the number of patients excluded because of the presence of osteoarthritis. In our opinion, restricting hemiarthroplasy to patients without detectable radiological signs of pre-existing osteoarthritis when treating femoral neck fracture would substantially limit its contribution to and value in routine clinical practice.

A major limitation of our study is the lack of radiologic follow-up, meaning that we were not able to characterize radiologic cartilage wear and the frequency of acetabular erosions in this cohort. Nevertheless, after 12 months all patients were clinically examined and hip function was assessed by the HHS, which is a widely accepted and validated outcome measure. Only patients with persistent hip pain received follow-up radiographs, and no hip was revised as a result of radiological signs of acetabular erosion. Furthermore, Figved et al. found no correlation between cartilage wear and functional outcome in their recent cohort study [21]. Interestingly, van Egmond et al. reported a 96 % survival after 15 and 60 % after 20 years in young patients with osteonecrosis or a tumour of the proximal femur, and hypothesized that bipolar hemiarthroplasty can even be can be superior to a THA in young patients [22]

Another potential drawback of our study might be the follow-up time of 12 months. When compared with cohorts of patients undergoing elective THA for osteoarthritis, the relatively high mortality rates and patients’ steadily increasing frailty limit the feasibility of a long-term functional follow-up in the population with proximal femoral fracture. Interestingly, a recent radiostereometric analysis showed progressive erosion around the femoral head between 3 and 12 months after surgery, but no further relevant cartilage wear [21], indicating that cartilage degeneration may plateau after 1 year.

Despite the relatively large size of our prospectively observed cohort, only 38 patients in group 1 and 13 patients in group 2 were available at final follow up. Thus, the sample size respresents another important limitation of the present study. Here, the future acquisition of multicentre registry data could result in a higher power of the explorative statistical analysis.

Pre-operative hip osteoarthritis was evaluated according to Kellgren and Lawrence’s criteria. The limitations of this classification system have been extensively investigated and discussed [23], yet it remains the standard means of assessing the extent of osteoarthritis of the hip on radiographs, is easy to interpret and allows comparison with other cohorts. To minimize the possible effect of the limited diagnostic precision of the Kellgren and Lawrence classification, we grouped the cohort into patients with grade 0 to 2 changes as having absent or moderate osteoarthritis, and patients with grade 3 and 4 changes as having severe disease.

The main strengths of our study are the stringent follow-up routine, the homogenous and highly standardized treatment algorithm and the use of three highly validated and reproducible functional outcome measures.

## Conclusions

We found no correlation between the grade of pre-traumatic osteoarthritis of the hip and the short-term functional outcome in elderly patients with femoral neck fracture treated by hemiarthroplasy. Furthermore, the existence of osteoarthritis did not influence the post-operative complication or revision rate. Taken together, radiologic evidence of osteoarthritis of the hip may not reliably inform the choice between hemiarthroplasy and THA in elderly patients with femoral neck fracture. Prospective clinical trials establishing the outcome of cohorts with osteoarthritis treated with both implant types are required.

## Abbreviations

ASA: American society of anaesthesiologists
FU: Follow up
HSS: Harris hip score
MMSE: Mini–mental state examination
OA: Osteoarthritis
TUG: Timed up and go
THA: Total hip arthroplasty
